# Power Production and Degradation of Pesticide Wastewater Through Microbial Fuel Cells with the Modified Activated Carbon Air Cathode by Hollow-Carbon and Carbon-Encapsulated Structures

**DOI:** 10.3390/molecules29235675

**Published:** 2024-11-30

**Authors:** Xueli Zhang, Linhui Jia, Yu Liu, Ziqi Wang, Jumiao Qin, Qiuhong Wang, Xiao Zhao, Ming Zhong, Jianfeng Lang, Guangri Xu, Yanbing Wu, Chengxing Cui

**Affiliations:** 1 School of Resources and Environment, Henan Institute of Science and Technology, Xinxiang 453000, China; zhangxueli@hist.edu.cn (X.Z.);; 2 School of Chemistry and Chemical Engineering, Henan Institute of Science and Technology, Xinxiang 453000, China; xugr70@163.com; 3 College of Environmental Science and Engineering, Nankai University, Tianjin 300071, China

**Keywords:** microbial fuel cell, oxygen reduction reaction, metal-organic framework, pesticide degradation

## Abstract

Microbial fuel cell (MFC) can degrade pesticide wastewater and recovery energy simultaneously, and the activated carbon (AC) air cathode has great prospects for practical application. However, insufficient active sites and the limitation of multi-step electron transfer for oxygen reduction reaction (ORR) requires that AC should be modified by highly efficient electrocatalysts. Herein, busing the confinement effect of carbon-encapsulated metal and hollow carbon, we designed a unique ORR catalyst of Fe-Fe_3_O_4_-NC through utilizing the 2D leaf-like nanoplates of Zn-ZIF-L to load Prussian blue (PB) particles. The volatilization of low-boiled Zn and the catalysis of iron compounds led to the formation of confined walls of hollow carbon shell and carbon-encapsulated Fe/Fe_3_O_4_ particles on N-doped carbon substrate. Multivalent iron, a large surface area (368.11 m^2^·g^−1^), N doping, a heterojunction interface, and the confinement effect provided all the Fe-Fe_3_O_4_-NC-modified AC air cathodes with excellent ORR activity. The optimal samples of AC-Fe-Fe_3_O_4_-NC-3 achieved a peak power density of 1213.8 mW·m^−2^, demonstrating a substantial 82.8% increase over that of the bare AC. Furthermore, its efficiency in glyphosate removal reached 80.1%, surpassing the 23.2% of the bare AC. This study offers new ideas in constructing composite confined structures and the as-designed Fe-Fe_3_O_4_-NC is a promising modification candidate for the commercial adoption of AC air cathodes.

## 1. Introduction

Pesticide production wastewater is one of the main factors that cause severe chemical industrial pollution [1,2]. For example, glyphosate (N-phosphonomethyl glycine, (HO)_2_P(=O)-CH_2_-NH-CH_2_-COOH, C_3_H_8_NO_5_P) is a widespread, globally applied pesticide due its minimal harm to non-target organisms and strong efficacy against deeply rooted weeds, but it produces a large quantity of wastewater and contains a high concentration of COD and organic pollutants, posing a serious threat to surface soil and groundwater [3]. The output of glyphosate pesticides is about 11,180,000 tons per year all over the word, but the efficiency of its treatment technologies such as MVR technology, multi-effect evaporation, advanced oxidation, and electrodialysis needs to be improved [4,5], among which some may be extremely energy intensive or may cause secondary pollution. Fortunately, glyphosate is easily biodegradable, and it is worth noting that the high organic pollutants include a large amount of organic chemical energy of 1.4 × 10^7^ J/kg COD [6], which possess great potential to be recovered into green energy. Therefore, special technologies for degrading pesticide wastewater and recovering energy are urgently required.

Microbial fuel cells (MFCs) can degrade organic sewage and produce electricity simultaneously and recognized as being a novel environmentally friendly energy-conversion technology [7,8]. However, the weak power generation of MFCs poses a bottleneck for its industrial development. Generally, the output electricity of MFC is notably influenced by multiple factors, such as electrode materials, microorganism species, reactor configurations, and operational parameters. Among these factors, electrode materials play a crucial role in determining the power output efficiency. In MFCs, electrons released by electrogenic microorganisms after their degradation to organic pollutants are commonly accepted by oxygen from the air at the cathode, thus facilitating oxygen reduction reaction (ORR) [9,10]. As a consequence, following the four-electron ORR pathway is favorable for MFCs in achieving energy conversion because it acquires a high output potential and turns organic pollutants into CO_2_ and H_2_O thoroughly [11]. However, the four-step electron transfer involves a sluggish kinetics, and therefore highly active electrocatalysts are required to facilitate e^−^ acceptance. In this regard, it is scientific and effective to modify cathodes with instrumental catalysts to improve the performance of MFCs [12]. Moreover, in a single-chamber configuration, which is well acknowledged for its low cost and simplicity, the survival of microorganisms and interference of organic matters require a strict selection of catalysts. Specifically, the low-cost activated carbon (AC) is easy to obtain and appears to be most suitable for large-scale applications. However, the activity of AC is suboptimal for MFCs due to its poor ORR selectivity and limitations in powerful active sites for bond O-intermediates [13,14,15]. Therefore, modifying the AC cathode with highly efficient electrocatalysts is a key step to developing MFCs toward practical application and remains a challenging research hotspot.

The critical point of ORR catalysis is ensuring that enough O_2_ and H^+^ at the active site receive the incoming electrons and produce H_2_O quickly. Therefore, the origin of designing ORR electrocatalysts lies in boosting the mass transfer, the dispersion of active sites, and the activity of reaction sites [16,17]. In this regard, porous carbon can usually provide an excellent surface structure for O_2_/H^+^ to diffusion through meso/macropores and react on the micropore surface [18,19]. In particular, the hollow carbon structure can shorten the electron transfer distance and confine the reactants spatially to obtain a high concentration for fast kinetics [20,21]. However, pure carbon materials have been frequently reported to follow a two-electron oxygen reduction pathway, which exhibits weaker activity toward four electrons [22]. In recent years, efforts have been devoted to modifying carbon materials by encapsulating active metals to facilitate oxygen reduction in favor of four-electron transfer [23,24,25]. The rearrangement of interface charges between carbon-encapsulated metals regulates the adsorption energy of oxygen intermediates on active sites, and the carbon shell can effectively prevent the dissolution of metal ions, thereby increasing the stability of the catalyst. Especially for the N-doped carbon, N incorporation can not only anchor the inner Fe atoms but also break the charge distribution on the carbon surface and thus attract more reactants to adsorb on the active sites. Zhu et al. revealed that when Fe atoms were introduced into the nonplanarity of N-C, the binding energy of *OOH could be enhanced and the ORR pathwaycould thus be tailored into 4e^−^ [26]. Zheng et al. confirmed that the core-shell Co-CrN@C exhibited almost ideal four-electron reduction of oxygen due to the high-degree graphitization of the carbon shell and intrinsic activity of Co-Cr sites [27]. Liu discovered that the -OOH* dissociation of the 4e^−^ pathway predominated when Mn-N_4_ was present between FeMn atomic clusters and the N-doped carbon shell [28]. Additionally, the structure of carbon-encapsulated metal has been simulated as an endohedral metallofullerenes model by first-principles study, with the order of ORR efficiency for metal-incorporated carbons being as follows: Fe > Co > Mn > Cu > Ni [29]. Obviously, among these metals, Fe stands out as the optimal dopant due to it having the highest catalytic activity and the lowest cost. The above findings indicate that designing unique structures of hollow and carbon-encapsulated Fe species is a viable approach for using carbon materials to achieve complete oxygen reduction for fuel cells.

Zeolite imidazole frameworks (ZIFs) are a preferred kind of metal-organic framework (MOF) precursors for deriving porous carbon catalysts, which have a diversity of components and good thermal and chemical stability [30,31]. Especially for Zn-ZIF, owing to the thorough decomposition of organic ligands and Zn nods, it has displayed almost the highest surface area among ZIF-derived carbon materials in offering a large surface reaction region [32,33]. However, the only micropores and weak carbon active sites in its structure hinder its mass diffusion and decline of ORR catalysis [32]. Therefore, improving the pore structures and making modifications on the oxygen reduction pathway for Zn-ZIF-derived carbon may prove valuable. In this regard, carbon-encapsulated Fe particles derived from Prussian blue (PB) precursor has attracted much attention, due to its low cost, simple preparation, and amenability to four-electron ORR [34,35]. The local electron population at the interface of Fe cores and N-doped carbon shell is rearranged due to their differences in energy and electronegativity, which activate Fe sites and simultaneously protect Fe from leakage. However, owing to the high Fe content in PB, the calcined Fe particles in PB-derived catalysts severely block the pores, which results in poor specific surface area, low nitrogen content, and the agglomeration of active sites. Therefore, a high porous matrix is needed for PB-derived catalysts to disperse the highly active Fe species. Consequently, considering the advantages and disadvantages of PB and ZIF, it is not difficult to discern that coupling PB with ZIF can compensate for each other’s shortcomings in the design of a high-performance ORR catalyst. PB can provide active iron species for ZIF, and ZIF can offer a broad carbon matrix for dispersing PB active sites. At the same time, during the pyrolysis process, iron atoms can catalyze amorphous carbon to form possible hollow or graphite carbon shells coated with iron particles, which can accelerate electrolyte penetration, oxygen diffusion, and catalyst stability.

Herein, a 2D leaf-like microplate of zinc-based zeolite imidazole frameworks(Zn-ZIF-L) was selected to load PB, and the as-designed electrocatalyst of N-doped carbon-encapsulated Fe-Fe_3_O_4_ particles with confined hollow carbon shells (Fe-Fe_3_O_4_-NC) was obtained after thermal treatment. Physical characterizations confirmed that Fe-Fe_3_O_4_-NC possessed a plentiful amount micropores and mesopores compared with pure Zn-ZIF-L-derived NC and PB-derived PB-C. The carbon matrix endowed the Fe-Fe_3_O_4_-NC with an improved surface structure compared with that of PB-C. The polyvalent of Fe^0^ and Fe^II^/Fe^III^ contributed to the acceptance of electrons and the regeneration of active sites. As a result, the Fe-Fe_3_O_4_-NC displayed excellent electrochemical ORR activity, and when applied to MFC, the AC air cathode doped with Fe-Fe_3_O_4_-NC achieved a higher output power density and COD removal rate than those of pure activated carbon. Our study provides insights into the design of composite confined catalysts based on ZIF-derived carbons and paves the way for the application of AC-based MFC cathodes.

## 2. Results and Discussion

### 2.1. Physical Characterization Results and Analysis

**Figure 1:**
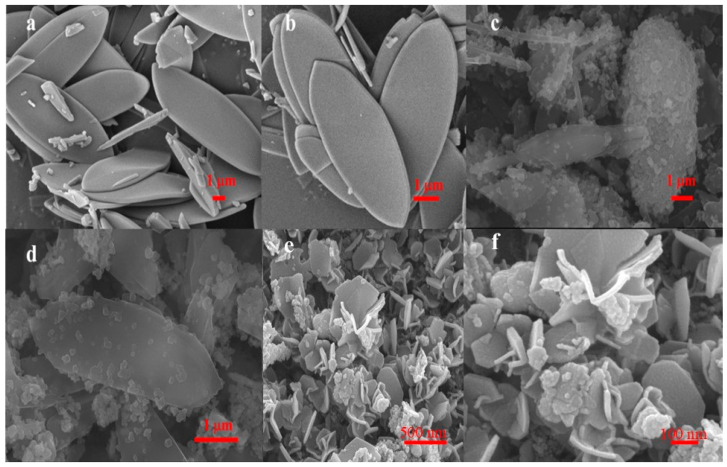
SEM images of (**a**,**b**) Zn-ZIF-L, (**c**,**d**) Zn-ZIF-L@PB. and (**e**,**f**) Fe-Fe_3_O_4_-NC.

The ORR performance was closely related to factors such as the intrinsic activity and dispersibility of active sites, O_2_ diffusion, and the penetration of electrolytes. In this study, Zn-ZIF-L was designed to load PB precursor and to better disperse Fe-based active sites on the derived carbon matrix. Scanning electron microscopy (SEM, Figure 1a,b) indicated that the as-synthesized Zn-ZIF-L exhibited a leaf-like 2D structure with a smooth surface, measuring approximately 7 μm in length and 3 μm in width. It can be observed from the X-ray diffraction (XRD) peaks of the Zn-ZIF-L (shown in the following section of Figure 3a) were essentially the same as those of the simulated XRD patterns based on the crystal structure and the ZIF-L reported in the literature [36], indicating its successful synthesis. It should be noted that the choice of 2D Zn-ZIF-L as the substrate MOF was justified and reasonable because its synthesis process omitted harmful organic solvents that are typically indispensable for the fabrication of most MOFs. Moreover, the 2D Zn-ZIF-L could contribute a broad surface to capture PB crystalline for better composition. After compositing with PB under ultrasound, the Zn-ZIF-L@PB precursor was fabricated. The SEM images of Zn-ZIF-L@PB (Figure 1c,d) further proved that the leaf-structured Zn-ZIF-L was covered with some nucleated PB particles being distributed on the surface as expected.

**Figure 2:**
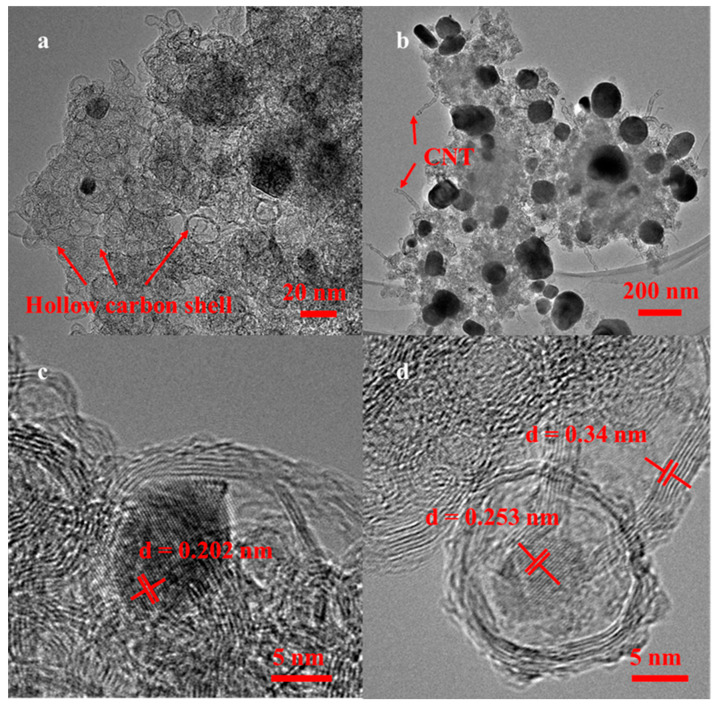
(**a**,**b**) TEM images and (**c**,**d**) HRTEM images of Fe-Fe_3_O_4_-NC.

After carbonization, the SEM images of Zn-ZIF-L@PB-derived Fe-Fe_3_O_4_-NC (Figure 1e,f) presented a flower-shaped structure, which was composed of nanosheets and dotted nanoparticles. As is well-known, the carbonized derivatives of MOFs typically retain the morphology of their precursors [37]. However, the morphological rearrangement of Zn-ZIF-L substrate from 2D nanoplates to a 3D flower during thermal treatment was clearly demonstrated in this study. The reason might be that although the Zn-ZIF-L could provide a carbon matrix for PB derived iron-based particles, its steady-state is a rhombic dodecahedron rather than a 2D leaf shape, resulting in a carbon fusion effect and nanosheet deformation during pyrolysis [38]. A transmission electron microscope (TEM) test was conducted to further observe the microstructure and lattice fringes of the Fe-Fe_3_O_4_-NC. The results showed that PB-derived, Fe-based particles embedded in the porous carbon matrix (Figure 2a,b), which was constituted of hollow carbon shells and seldom of carbon nanotubes (CNTs). The hollow carbon shell might be formed by the volatilization of zinc in the precursor when pyrolysis temperature surpassed 1000 °C due to zinc particle gasification [39], and the formation of CNTs was closely related to the catalysis of Fe species on carbon matrix [40]. The enlarged high-resolution TEM (HRTEM) indicated that the Fe particles were encapsulated by multi-walled graphene layers (Figure 2c,d). The inside lattice fringes with an interplanar distance of 0.202 nm corresponded to the (110) planes of Fe, and the lattice fringes with an interplanar distance of 0.253 nm corresponded to the (311) planes of Fe_3_O_4_. The outside lattice spacings of carbon shells were measured to be 0.34 nm, which corresponded to the (002) planes of graphite carbon. It was reported that Fe^III^ underwent reduction to form an metal-iron-on-carbon matrix, which subsequently interacted with oxygen to yield iron oxides [41]. This process is important for the subsequent catalytic effect of the formed iron and iron oxides on nearby carbon atoms, leading to the formation of graphene layers [40]. Consequently, with the volatilization of Zn particles, a confinement structure emerged where nitrogen-doped graphene layers served as the outer shell, enveloping a hollow structure or the iron/iron oxide core.

**Figure 3:**
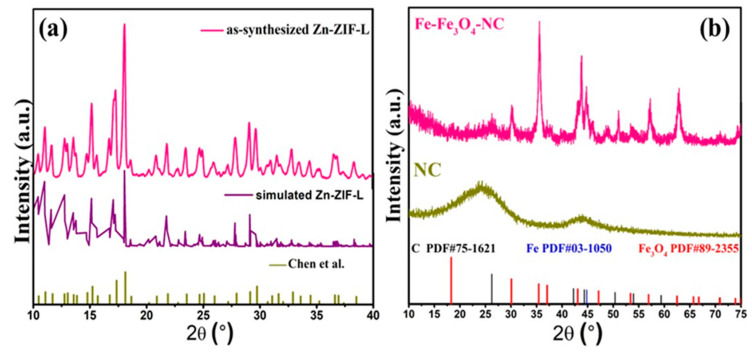
XRD patterns of (**a**) as-synthesized, simulated, and reported Zn-ZIF-L [36]. (**b**) NC and Fe-Fe_3_O_4_-NC.

The crystal compositions of the synthesized catalysts were further revealed by the XRD analysis (Figure 3b). In the case of NC, the diffraction peaks observed at 26.2° and 44.3° corresponded to the (002) and (101) planes of graphite carbon (PDF#75-1621), respectively. This suggested that it served as the carbon matrix backbone in the composite Fe-Fe_3_O_4_-NC. For Fe-Fe_3_O_4_-NC, the distinct diffraction peaks observed at 30.0°, 35.4°, 43.0°, 56.9°, and 62.5° corresponded to the (220), (311), (400), (511), and (531) planes of Fe_3_O_4_ (PDF#89-2355), respectively, and the peak at 44.8° corresponded to the (110) plane of Fe (PDF#03-1050). Moreover, the unique diffraction peaks belonging to NC turned out to be sharper and more distinct, which was ascribed to the presence of CNTs and graphite carbon shell. This higher graphitization meant fast electron transport on the surface of Fe-Fe_3_O_4_-NC.

**Figure 4:**
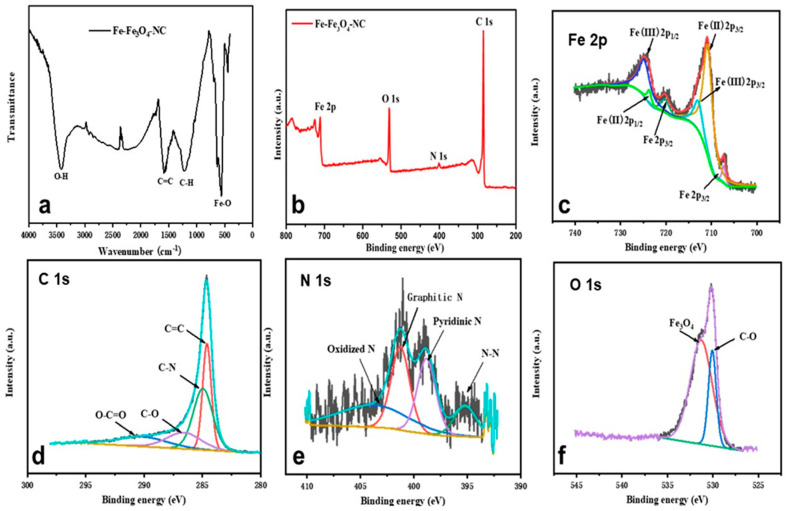
(**a**,**b**) FTIR and XPS spectra of Fe-Fe_3_O_4_-NC. (**c**) Core-level spectra of Fe 2p. (**d**) Core-level spectra of C 1s. (**e**) Core-level spectra of N 1s. (**f**) Core-level spectra of O 1s.

In order to have a more detailed understanding of the functional groups and chemical bonds of the catalyst material, FTIR testing on Fe-Fe_3_O_4_-NC was conducted. The results are shown in Figure 4a. The peak at 586 cm^−1^ in the spectrum was attributed to the stretching and bending vibrations of the Fe-O bonds in the tetrahedral and octahedral positions of Fe_3_O_4_ and the peak at 1579 cm^−1^ corresponded to the in-plane ring vibrations of carbon–carbon double bonds [42]. The vibrations at 1217 cm^−1^ and 3421 cm^−1^ were associated with C-H and O-H [41], and this analysis was consistent with the XRD results.

XPS tests were conducted to further explore the surface composition of the catalyst. The XPS elemental analysis (Figure 4b) revealed the presence of Fe, O, N, and C in the Fe-Fe_3_O_4_-NC sample, with atomic composition percentages of 6.99% for Fe, 12.34% for O, 1.79% for N, and a dominant 78.88% for C, respectively. Evidently, the quantities of Fe species were notably inferior to that of C, strongly suggesting that the Fe-Fe_3_O_4_-NC surfaces were largely enveloped by graphene layers. Furthermore, the absence of Zn signals in the elemental survey indicated that Zn was thoroughly eliminated through high-temperature carbonization. Specifically, the Fe 2p spectrum was analyzed (Figure 4c), and the prominent peaks situated at 707.3 eV and 720.1 eV were confirmed to be metallic iron [13], consistent with findings from XRD and HRTEM analyses. Additionally, the peaks detected at 710.8 eV and 723.7 eV matched with Fe(II) 2p3/2 and Fe(II) 2p1/2, respectively, and the peaks at 712.9 eV and 724.8 eV corresponded to Fe(III) 2p3/2 and Fe(III) 2p1/2, respectively [43]. Particularly, the peak at 710.6 eV signified N-coordinated iron species (Fe-N_x_) [13], which were commonly recognized as key catalytic sites in the ORR. Moreover, the valence cycle between Fe(III) and Fe(II) was beneficial for ad/de-sorption of O-intermediates. It was reported that Fe(III)-N_4_ tends to bond oxygen species, but the too-large amount of adsorption energy blocks the initial adsorption of O_2_ until the site is reduced to the Fe(II)-N_4_ form with the hydroxyl species removed [44]. In the C 1s spectrum depicted in Figure 4d, four fitted peaks were obtained, which were attributed to C=C at 284.6 eV, C-N at 285.0 eV, C-O at 286.5 eV, and O-C=O at 290.1 eV, respectively [13,44]. Here, C=C signifies the fundamental graphitic lattice, while the emergence of C-O was potentially a result of surface oxidation processes. Notably, the detection of C-N bonds implied successful nitrogen doping into the carbon framework. The high electronegativity of N embedded positive charges on adjacent carbon atoms, thereby creating reactive sites for oxygen-containing intermediate interaction. In Figure 4e, the four fitted peaks of N 1s at 395.1 eV, 398.8 eV, 401.2 eV, and 403.7 eV correspond to N-N (12.92%), pyridine-N (17.17%), graphite-N (30.11%), and nitric oxide (29.79%). Evidently, the content of graphitic nitrogen was the highest, which played an important role in promoting the oxygen reduction reaction [45]. According to the analysis of the fitted spectrum of O 1s in Figure 4f, the peaks at 530.0 eV and 531.3 eV in the spectrum corresponded to C-O and Fe-O [42], with respective contents of 24.50% and 75.50%, indicating a main form of Fe_3_O_4_ in the catalyst material. According to previous studies, transition metal oxides (Co_3_O_4_, MnO_2_, and Fe_3_O_4_) have a significant promoting effect on the ORR due to the oxidation–reduction interaction between multivalences [46].

**Figure 5:**
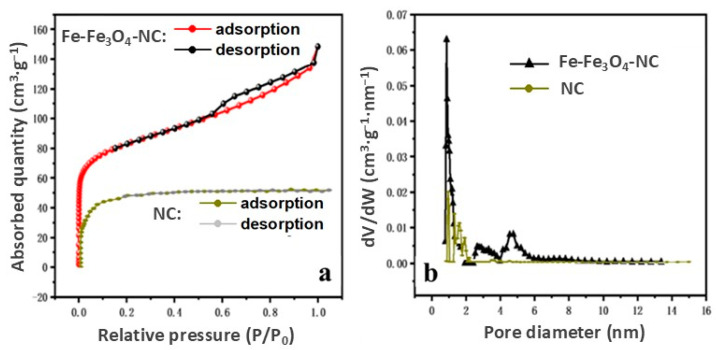
(**a**) Adsorption and desorption isotherm of Fe-Fe_3_O_4_-NC and NC. (**b**) Pore size distribution.

N_2_ adsorption–desorption measurements were analyzed to explore the changes in specific surface area and pore structure information before and after compositing. From Figure 5a, it is evident that Fe-Fe_3_O_4_-NC exhibited a type-IV isotherm, a characteristic feature indicative of a mesoporous structure. Figure 5b and Table S1 indicate that its pore diameters were centered on 1.1, 2.3, and 4.3 nm, which are consistent with those of PB-C (Figure S1). However, the hysteresis loop of PB-C was much smaller owning to its poor surface area of 29.97 m^2^·g^−1^ compared with that of Fe-Fe_3_O_4_-NC (368.11 m^2^·g^−1^). The N_2_ adsorption–desorption curves of NC are a typical-I isotherm, and the pore diameter mainly centered on 1.5 nm, which indicated a micropore structure. Unexpectedly, its surface area was smaller (122.9 m^2^·g^−1^) than that of Fe-Fe_3_O_4_-NC. Different from conventional carbon composite materials, the 2D of NC plane underwent structural deformation due to the carbon fusion effect during heating (Figure S2) and reduced its surface area dramatically [47]. However, the combination of PB and Zn-ZIF-L slowed down the collapse of the Zn-ZIF-L structure during the pyrolysis process. Therefore, it could be inferred that the synergistic effect between PB and Zn-ZIF-L in the precursor of Fe-Fe_3_O_4_-NC overcame the disadvantage of poor surface structure of PB-C and NC themselves. Additionally, the total pore volume for Fe-Fe_3_O_4_-NC was 0.14 cm^3^·g^−1^ which was the same as that of NC and was higher than that of PB-C (0.038 cm^3^·g^−1^). This suggests that the loaded Fe species were dispersed well on NC and did not block pores but enhanced mesopores, as can be seen in Table S1. For electrocatalysts, the coexistence of micropores and mesopores facilitated surface reactions because micropores provided active sites for electron transfer while the mesopores acted as channels for oxygen transport [43].

Given the above findings, the as-synthesized Fe-Fe_3_O_4_-NC, with unique features of carbon-encapsulated Fe and Fe oxide particles, hollow carbon shell, CNTs and coupled micro/mesopores, contributed spatially confined catalysis and mass transport for ORR. In addition, the carbon shell could anchor metal nanoparticles and prevent leakage, which enhanced the stability of ORR.

### 2.2. Electrochemical Analysis

#### 2.2.1. ORR Catalytic Activity

**Figure 6:**
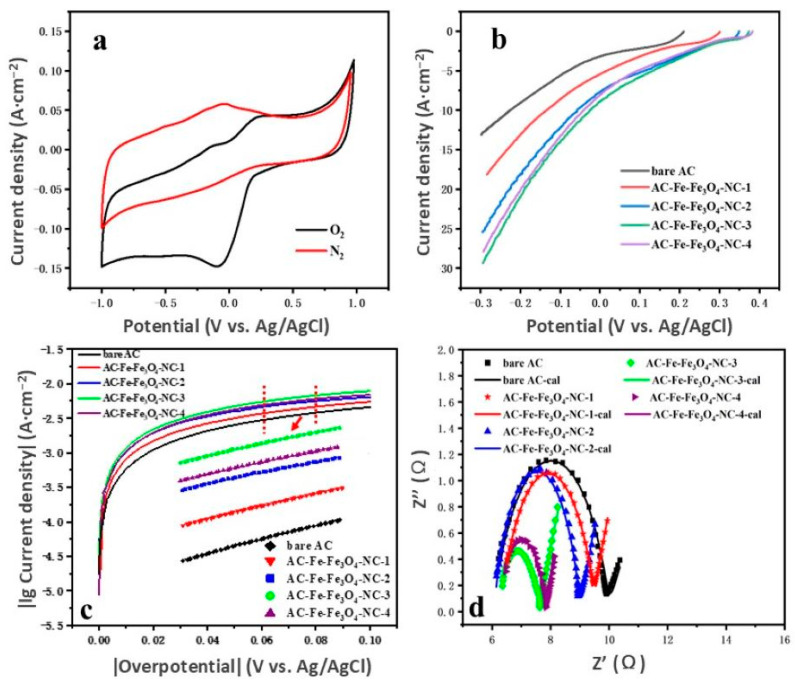
(**a**) CV curves of Fe-Fe_3_O_4_-NC. (**b**) LSV curves of Fe-Fe_3_O_4_-NC-doped AC cathodes with different proportions. (**c**) Tafel curves (the insert shows the fitting lines) and (**d**) EIS fitting curves.

Based on preceding discussions, the carefully engineered Fe-Fe_3_O_4_-NC, boasting superior electronic and surface structures, could serve as a highly effective ORR catalyst for enhancing the performance of the AC air cathode. Initially, the ORR performance of the as-fabricated Fe-Fe_3_O_4_-NC was examined through cyclic voltammetry (CV) tests under oxygen-saturated and nitrogen-saturated conditions in a neutral PBS solution (50 mM). In an O_2_-rich environment, a distinct oxygen reduction peak appeared, but it vanished when the solution was saturated with N_2_, indicating the selective response of Fe-Fe_3_O_4_-NC to ORR (Figure 6a).

Based on above findings, Fe-Fe_3_O_4_-NC was doped in AC air cathodes with different proportions for further evaluation of ORR activity via LSV tests. Figure 6b indicates that the current densities of all the Fe-Fe_3_O_4_-NC-modified AC air cathodes were much higher than that of the bare AC air cathode. At −0. 3 V, the current densities corresponding to the air cathodes with different doping ratios were as follows: AC-Fe-Fe_3_O_4_-NC-3, (29.35 mA·cm^−2^) > AC-Fe-Fe_3_O_4_-NC-4 (27.89 mA·cm^−2^) > AC-Fe-Fe_3_O_4_-NC-2 (25.43 mA·cm^−2^) > AC-Fe-Fe_3_O_4_-NC-1 (18.12 mA·cm^−2^) > bare AC (13.10 mA·cm^−2^). This suggests that the optimal doping ratio was 50%, which increased near to 124% as compared to the bare AC air cathode.

#### 2.2.2. Catalytic Kinetic Analysis of ORR

The oxygen reduction kinetics of the cathode catalyst was estimated by exchange current density (*i*_0_). This offered valuable insights into the efficiency of electron transfer, with a higher *i*_0_ indicating a more effective transfer process. In Figure 6c, the Tafel plots exhibit an initial steep increase followed by a gradual slowdown, with a marked divergence evident at overpotentials exceeding 20 mV. This behavior signified unsynchronized electron transfer of the different AC air cathodes. The fitting lines (the insert in Figure 6c) with linear regression indicate that the coefficients (Table 1) of all the AC air cathodes surpassed 0.99, and the order of *i*_0_ was as follows: AC-Fe-Fe_3_O_4_-NC-3 (29.09 × 10^−4^ A·cm^−2^) > AC-Fe-Fe_3_O_4_-NC-4 (26.46 × 10^−4^ A·cm^−2^) > AC-Fe-Fe_3_O_4_-NC-2 (25.70 × 10^−4^ A·cm^−2^) > AC-Fe-Fe_3_O_4_-NC-1 (18.76 × 10^−4^ A·cm^−2^) > bare AC (14.07 × 10^−4^ A·cm^−2^). For AC-Fe-Fe_3_O_4_-NC-3, it increased nearly 106.8% compared to the bare AC air cathode, indicating that the oxygen reduction kinetics of the air cathode were greatly improved.

Additionally, electrochemical impedance played a pivotal role in assessing the electrochemical process kinetics. Electrochemical impedance spectroscopy (EIS) reflected the charge transfer and mass transfer on the electrode surface during oxygen reduction. As shown in Figure 6d and the fitting results in Table 2, the equivalent circuit (Figure S3) accounted for three resistance components: ohmic resistance (R_0_), diffusion resistance (R_d_), and charge transfer resistance (R_ct_). R_0_ refers to the intrinsic resistance of MFC reactors, and in this study, the R_0_ values of various Fe-Fe_3_O_4_-NC modified AC air cathodes were almost same (at about 6.1 Ω). Moreover, the R_d_ of the bare AC was 2.66 Ω, but the other air cathodes modified by Fe-Fe_3_O_4_-NC exhibited smaller values. Typically, R_d_ served as a crucial indicator for evaluating the oxygen and ion exchange rate between an electrode and the electrolyte. Li and Wang et al. reported that mesoporous structures enhanced ion transfer and oxygen diffusion, thereby reducing R_d_ [43,48]. Figure S4 shows that the adsorption–desorption isotherm of the as-used AC was type-I, which was indicative of microporous structure. As depicted in Table S1, Fe-Fe_3_O_4_-NC mainly contained mesopores, so the addition of more Fe-Fe_3_O_4_-NC triggered the decrease of R_d_, but excessive Fe-Fe_3_O_4_-NC might cause an increase of R_d_ due to the reduced surface area. R_ct_ was an indicator of electron transfer on the electrode–electrolyte interface. Figure 6d and Table 2 suggest a descending order of R_ct_ values for various air cathodes: bare AC (3.93 Ω) > AC-Fe-Fe_3_O_4_-NC-1 (2.90 Ω) > AC-Fe-Fe_3_O_4_-NC-2 (2.81 Ω) > AC-Fe-Fe_3_O_4_-NC-4 (1.65 Ω) > AC-Fe-Fe_3_O_4_-NC-3 (1.43 Ω). Obviously, all Fe-Fe_3_O_4_-NC-modified samples displayed lower R_ct_ compared to the unmodified AC, and AC-Fe-Fe_3_O_4_-NC-3 achieved the minimum, implying that Fe-Fe_3_O_4_-NC-3 accelerated ORR kinetics. Consequently, combining the above three resistances, the order of the total resistance Rt of each cathode was as follows AC-Fe-Fe_3_O_4_-NC-3 (9.67) > AC-Fe-Fe_3_O_4_-NC-4 (10.00) > AC-Fe-Fe_3_O_4_-NC-2 (11.24) > AC-Fe-Fe_3_O_4_-NC-1 (11.76) > bare AC (12.68). The minimum resistance value has decreased by 42.9% compared to the bare AC air cathode, and this result was consistent with the Tafel findings.

**Figure 7:**
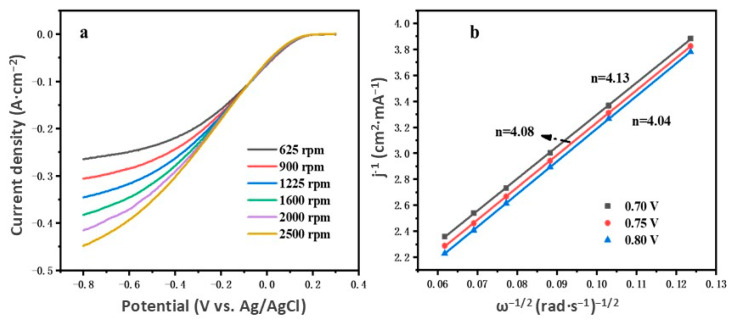
(**a**) RDE curves of Fe-Fe_3_O_4_-NC. (**b**) K-L plots.

To further validate the in-depth kinetics and oxygen reduction mechanism of the Fe-Fe_3_O_4_-NC, RDE curves were conducted at different rotation speeds (Figure 7a). The ORR pathway and the number of electron transfer were elucidated using the Koutecky–Levich (K-L) equation (Figure 7b, details are provided in the Supplementary Materials). The oxygen reduction processed mainly followed two distinct pathways. One was a two-electron process that was characterized by incomplete oxidation, leading to reduced energy conversion efficiency. Conversely, another pathway involved a four-electron reduction and was considered to be a comprehensive ORR process which yielded H_2_O as the terminal production. Therefore, the four-electron pathway was highly advantageous for microbial fuel cells in significantly enhancing the efficiency of energy conversion. As shown, the corresponding number of electron transfers was between 4.04 and 4.13, indicating that the ORR process of the Fe-Fe_3_O_4_-NC was driven by the four-electron pathway and also revealed that FFC/NG was a preeminent ORR catalyst for converting more chemical energy in wastewater into electrical energy.

#### 2.2.3. Power Generation and Glyphosate Removal of MFC

**Figure 8:**
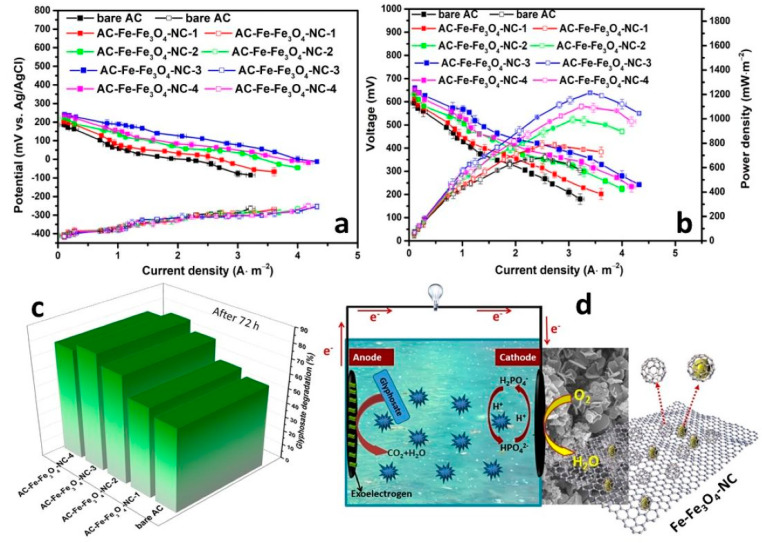
**(a)** Polarization curves. (**b**) Power density curves. (**c**) Glyphosate degradation efficency. (**d**) The diagram of ORR on Fe-Fe_3_O_4_-NC in MFC.

The as-designed AC air cathodes modified by Fe-Fe_3_O_4_-NC were equipped with carbon felt anode for assembling the MFC reactors. The simulated glyphosate pesticide wastewater was used to domesticate microorganisms until the output voltage became stable. The polarization curves and power density curves were measured to evaluate the performance of MFCs. Figure 8a shows that the anode voltage change of each MFC was basically the same, while substantial variations were observed in cathode voltages, indicating that the cathode was the chief influencer in electricity generation. This disparity can largely be attributed to discrepancies in the oxygen reduction activities and the dynamics of the air cathodes, as previously discussed. Notably, the cathode voltage pattern for the AC air cathodes involved a clear descending trend, in which AC-Fe-Fe_3_O_4_-NC-3 surpassed AC-Fe-Fe_3_O_4_-NC-4, followed by AC-Fe-Fe_3_O_4_-NC-2, AC-Fe-Fe_3_O_4_-NC-1, and finally, the unmodified AC-bare cathode. This sequence underscores the fact that Fe-Fe_3_O_4_-NC-modified AC cathodes consistently outperformed the bare AC cathode in terms of voltage output. Consequently, the total voltage and power density adhered to an identical ranking (Figure 8b). The maximum power density (MPD) of AC-Fe-Fe_3_O_4_-NC-1, AC-Fe-Fe_3_O_4_-NC-2, and AC-Fe-Fe_3_O_4_-NC-4 was 787.5 mW·m^−2^, 993.6 mW·m^−2^ and 1101.0 mW·m^−2^, respectively, and was 18.6%, 49.6%, and 65.8% higher than that of the bare AC (664.5 mW·m^−2^), respectively. The maximum power density (MPD) was 1213.8 mW·m^−2^ for AC-Fe-Fe_3_O_4_-NC-3, which was 82.8% higher than that of the bare AC. Moreover, the power output of the as-designed AC-Fe-Fe_3_O_4_-NC-3 was comparable or surpassed that pf many Fe-based or pesticide-water-related catalysts such as FePc/ACF (596.3 mW·m^−2^, glyphosate wastewater), FePc-rGO/ACF (1102 mW·m^−2^, glyphosate wastewater) [49], Fe_3_O_4_/Fe-N-C (831 mW·m^−2^, domestic wastewater) [44], Fe-Fe PBA-1000 AC air cathode (1108 mW·m^−2^, domestic wastewater) [13], graphite rods (1469 mW·m^−2^, pentachlorophenol wastewater) [50], and graphite felt (88 mW·m^−2^, 2,4,6-trichlorophenol wastewater) [51]. Furthermore, the efficiency of glyphosate removal was found to be the greatest for AC-Fe-Fe_3_O_4_-NC-3 (80.1%) as compared to 61.4%, 71.8%, and 75.5% for AC-Fe-Fe_3_O_4_-NC-1, AC-Fe-Fe_3_O_4_-NC-2, and AC-Fe-Fe_3_O_4_-NC-4, respectively, which were all higher than that of bare AC (56.9%) (Figure 8c and Figure S7). The above findings confirmed that Fe-Fe_3_O_4_-NC was an excellent dopant to improve the performance of the AC air cathode. As a whole, the superior performance of MFC using AC-Fe-Fe_3_O_4_-NC-3 can be attributed to the accelerated electron transfer and O-reactants reaction by the confined effect of hollow carbon and carbon-encapsulated Fe/Fe_3_O_4_ particles as well as the mesoporous structure (illustrated in Figure 8d).

## 3. Materials and Methods

### 3.1. Materials Synthesis

#### 3.1.1. Synthesis of Zn-ZIF-L

First, 1.18 g of Zn (NO_3_)_2_·6H_2_O (Aladdin) and 2.60 g of 2-methylimidazole (HMeIm) (Aladdin) were dissolved in 40 mL of deionized water, respectively. Subsequently, the Zn(NO_3_)_2_ solution was quickly poured into the 2-methylimidazole solution and stirred for 4 h. The color of the solution was gradually deepened and finally became rich milky white. Then, the white precipitate was collected by centrifugation and placed in an oven at 70 °C overnight, and Zn-ZIF-L powder was obtained after grinding.

#### 3.1.2. Synthesis of Zn-ZIF-L@PB

First, 0.80 g of Zn-ZIF-L powder and 0.70 g of Prussian blue (PB, Macklin) powder were dispersed in 20 mL of aqueous solution with ultrasonic oscillation for 30 min. It was then centrifuged at 10,000 rpm for 10 min, and the precipitate was collected. It was placed in an oven at 70 °C overnight and milled in a mortar to obtain the blue powder of Zn-ZIF-L@PB.

#### 3.1.3. Synthesis of Fe-Fe_3_O_4_-NC

**Figure 9:**
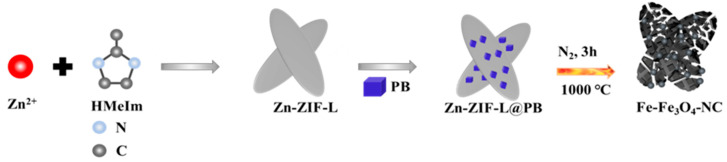
Synthesis scheme of Fe-Fe_3_O_4_-NC.

The synthesized Zn-ZIF-L@PB was first placed in a porcelain boat within a tubular furnace and was heated at 1000 °C for 3 h with a ramp rate of 5 °C·min^−1^ in a nitrogen atmosphere. The powder was then washed with deionized water and absolute ethanol several times and placed in a 70 °C oven overnight. The corresponding synthesis scheme of Fe-Fe_3_O_4_-NC is presented in Figure 9. Moreover, NC and PB-C were fabricated by the thermal treatment of pure Zn-ZIF-L and pure PB, respectively, in the same manner as that of Zn-ZIF-L@PB for comparison.

### 3.2. Air Cathode Fabrication

The air cathode of the microbial fuel cell was composed of three parts, namely a diffusion layer, a stainless-steel mesh. and a catalytic layer (Figure S5). To manufacture the diffusion layer, conductive carbon black powder was first soaked in an appropriate amount of anhydrous ethanol, and then the paste was added by 60 wt.% PTFE (the mass ratio of conductive carbon black to PTFE was 3:7). The paste was volatilized and was fully mixed with ultrasonic treatment for 30 min, and then it was rolled on one side of the stainless steel mesh and fixed by heating in a muffle furnace at 340 °C for 20 min. The catalytic layer was prepared by mixing the as-prepared catalytic material with activated carbon in a certain proportion to form a catalyst, and then an appropriate amount of anhydrous ethanol was added (the mass ratio of the catalyst and PTFE was 6:1). After ultrasonic treatment for 30 min, the paste was rolled on another side of the stainless-steel mesh. In this process, the mass proportions of Fe-Fe_3_O_4_-NC in the mixture of AC were 0%, 30%, 40%, 50%, and 60% and were denoted as bare AC, AC-Fe-Fe_3_O_4_-NC-1, AC-Fe-Fe_3_O_4_-NC-2, AC-Fe-Fe_3_O_4_-NC-3, and AC-Fe-Fe_3_O_4_-NC-4, respectively.

### 3.3. Assembly and Working of MFCs

The MFC configuration was assembled by four plexiglass plates with two gaskets, two washers, and electrodes (Figure S6). The plexiglass plate on the anode side was a sealing cover plate to ensure an anaerobic environment, and it was hollow on the cathode side to ensure direct contact with the air. MFC was fixed by four bolts and eight nuts, and a 1000 Ω resistance was loaded on to connect the anode and cathode. The volume of the MFC reactor was 28 mL, and the electrode area was 7 cm^2^.

To start up the MFCs, the culture solution and the sewage (1:1) were firstly added into the reactor, and then the output voltage of MFC was monitored by a data collector. The sewage mentioned above was taken from the secondary sedimentation tank of Xinxiang Luotuowan Sewage Treatment Plant. The culture medium was prepared by adding 0.2 g of anhydrous sodium acetate, 0.1 g of peptone, and 0.1 g of yeast powder into a 100 mL PBS solution (50 mM). In the initial stage of start-up, the volume ratio of sewage and nutrient solution was 1:1 for inoculation and culture. After a week, 2/3 of the solution was removed, and 1/3 of the sewage and 1/3 of the culture solution were injected into the reactor. This was repeated every two days until the voltage was stable and near the peak value. Biofilm was grown in the culture medium, and then the sodium acetate was gradually replaced by 30 mg/L of glyphosate (the pH in MFC was 6.7) until the output voltage became stable once again.

The information on material characterization, electrochemical measurements, and glyphosphate tests (Figure S8) is provided the in Supplementary Materials.

## 4. Conclusions

The AC cathodic catalyst is widely available and inexpensive, but its ORR activity needs to be modified by a highly efficient catalyst. In this study, using the advantages of hollow carbon and carbon-encapsulated Fe particles, we combined the complementary advantages of the PB and Zn-ZIF-L precursors and designed a unique catalyst of Fe-Fe_3_O_4_-NC. During synthesis, the micrometer-sized 2D plane of Zn-ZIF-L facilitated the sufficient adhesion of PB, carbon-encapsulated Fe/Fe_3_O_4_ was derived from the catalysis of iron atoms on the rearrangement of surrounding carbon atoms, and hollow carbon was formed due to the volatilization of Zn. Electrochemical analyses confirmed the superior ORR performance of all the Fe-Fe_3_O_4_-NC-modified air cathodes compared to that of the bare AC. The top-performing MFC with AC-Fe-Fe_3_O_4_-NC-3 achieved a maximum power density of 1213.8 mW m^−2^, marking an enhancement of 82.8% over that of the bare AC. Furthermore, the glyphosate removal of MFCs with AC-Fe-Fe_3_O_4_-NC-3 was 80.1%, which was 23.2% higher than that of the bare AC. Consequently, the as-designed Fe-Fe_3_O_4_-NC improved both the power generation and pesticide removal efficiency of AC air-cathode MFCs successfully, paving the way for promising large-scale practical applications.

## Figures and Tables

**Table 1:** Tafel curve fitting equation and exchange current density of AC air cathodes.

Cathode	Linear Equation	R^2^	*i*_0_ (×10^−4^ A·cm^−2^)
**bare AC**	y = 5.41398 × −2.8516	0.99787	14.07
**AC-Fe-Fe_3_O_4_-NC-1**	y = 4.91572 × −2.7268	0.99827	18.76
**AC-Fe-Fe_3_O_4_-NC-2**	y = 4.25058 × −2.5900	0.99473	25.70
**AC-Fe-Fe_3_O_4_-NC-3**	y = 4.58925 × −2.5362	0.99657	29.09
**AC-Fe-Fe_3_O_4_-NC-4**	y = 4.46443 × −2.5774	0.99707	26.46

**Table 2:** EIS fitting results of the Fe-Fe_3_O_4_-NC-modified AC air cathodes.

	Bare AC	AC-Fe-Fe_3_O_4_-NC-1	AC-Fe-Fe_3_O_4_-NC-2	AC-Fe-Fe_3_O_4_-NC-3	AC-Fe-Fe_3_O_4_-NC-4
R_0_ (Ω)	6.09	6.27	6.01	6.16	6.20
R_d_ (Ω)	2.66	2.58	2.41	2.02	2.15
R_ct_ (Ω)	3.93	2.90	2.81	1.48	1.65
R_t_ (Ω)	12.68	11.76	11.24	9.67	10.00

## Data Availability

The data presented in this study are available on request from the corresponding author.

## Supplementary Materials

The following supporting information can be downloaded at: https://www.mdpi.com/article/10.3390/molecules29235675/s1, Figure S1: Adsorption and desorption isotherm (a) and (b) pore size distribution of PB-C. Figure S2: The SEM image of Zn-ZIF-L-derived NC. Figure S3: Nernst equation fitting equivalent circuit diagram. Figure S4: (a,b) Nitrogen adsorption-desorption isotherm and pore size distribution of bared AC. Figure S5: Composition of MFC Cathode. Figure S6: Components of MFC reactor. Figure S7: The variation of glyphosate content with degradation time. Figure S8: UV-vis calibration curve of the standard glyphosphates solutions. Table S1: Comparison of surface area and pore information.
