# Supplementary material for: Power Production and Degradation of Pesticide Wastewater Through Microbial Fuel Cells with the Modified Activated Carbon Air Cathode by Hollow-Carbon and Carbon-Encapsulated Structures
Source: Molecules. 2024 Nov 30;29(23):5675. doi: 10.3390/molecules29235675 (PMC11643979; doi:10.3390/molecules29235675)
Supplement: Supplementary file 1 [file molecules-29-05675-s001.zip › molecules-3329668-supplementary.pdf]

Power Production and Degradation of Pesticide Wastewater Through Microbial Fuel Cells with the Modified Activated Carbon Air Cathode by Hollow-Carbon and Carbon-Encapsulated Structures

Xueli Zhang [1,2], Linhui Jia [1], Yu Liu [1], Ziqi Wang [1], Jumiao Qin [1], Qiuhong Wang [1], Xiao Zhao [1], Ming Zhong [3], Jianfeng Lang [1], Guangri Xu [2], Yanbing Wu [1,*] and Chengxing Cui [2,*]

1 School of Resources and Environment, Henan Institute of Science and Technology, Xinxiang 453000, China
2 School of Chemistry and Chemical Engineering, Henan Institute of Science and Technology, Xinxiang 453000, China
3 College of Environmental Science and Engineering, Nankai University, Tianjin 300071, China
* Correspondence: wybhist@126.com (Y.W.); chengxingcui@hist.edu.cn (C.C.)

Material characterization

Microstructure was characterized by scanning electron microscopy (SEM, ZEISS MERLIN Compact) and transmission electron microscopy (TEM, FEI TF30). Composition characterization was carried on the X-ray Diffraction (XRD, Ulitma IV with Cu Kα radiation), Raman spectrum (LabRAM HR Evolution, laser excitation 532 nm), Fourier transform infrared absorption (FTIR, Nicolet IS 10, 400-4000 nm) and X-ray photoelectron spectrometer (XPS, Thermo ESCALAB-250Xi). Nitrogen adsorption-desorption isotherms were obtained at 77.3 K using the (Quantachrome) SSA-7000.

Electrochemical measurements

Electrochemical tests were performed on a Princeton VersaSTAT3 electrochemical workstation in a typical three-electrode system with an Ag/AgCl

(saturated KCl solution) electrode as the reference electrode, Pt of 1 cm^2 as the counter electrode. The catalyst ink (5 $mg \cdot mL^{-1}$) were prepared by dispersing 5 mg of the obtained catalysts into the 1 mL solution containing 0.48 mL of deionized water, 0.48 mL of ethanol and 40 μL of 0.5 wt.% Nafion solution, followed by ultrasonication for 1 h. Then, 6 μL of the catalysts ink was dropped on a 4 mm diameter glassy carbon rotating disk electrode (RDE) to form a thin catalyst film, acting as the working electrode. The 50 mM PBS (pH=6.9) solution was employed as electrolyte, containing KCl, NH_4Cl, Na_2HPO_4, and $NaH_2PO_4 \cdot 2H_2O$ with concentrations of 0.13 $g \cdot L^{-1}$, 0.31 $g \cdot L^{-1}$, 4.09 $g \cdot L^{-1}$ and 3.32 $g \cdot L^{-1}$, respectively. LSV curves were conducted from 0.3 V to -0.8 V at a rotation rate of 1600 rpm. The potential range of RDE was set from 0.4 to − 0.8 V with a sweeping rate of 10 $mV \cdot s^{-1}$. The rotating speed was varied from 625 to 2500 rpm in an O_2-saturated PBS solution and the electron number was calculated from Koutecky-Levich equation.

Glyphosphates test

After the MFC process was completed, the reaction samples were analyzed for the concentration of glyphosphates. This was done by using a UV-Vis spectrophotometer (752N, Yidian, China) after filtration. This method has been reported previously in many literatures (J. power sources, 2021, 514, 230592; J. Mater. Sci., 2023, 58, 12569-12583; J. Alloy. Compd, 2023, 935, 168208; Chemosphere, 2024, 347, 140709). The UV-vis calibration plot for standard glyphosphates solutions (Fig.S2) as well as the equipment have been added in the revised supporting information, in which the R^2 was 0.99901 and displayed an excellent linear relationship.

Koutecky-Levich (K-L) equation

The electron transfer number (n) of the as-synthesized FFC/NG was calculated by the Koutecky-Levich equation.

$$1/j = 1/nAFkC_o - 1/\left(0.62nAF{D_o}^{2/3}\upsilon^{-1/6}C_o\omega^{1/2}\right)$$

where F is the Faraday constant (F = 96,485 C mol^{-1}), D_o is the diffusion coefficient of O_2 (D_o = 2.7 × 10^{-5} cm^2 s^{-1}), A is the area of the glass carbon electrode, C_o is the concentration of O_2 dissolved in the electrolyte (C_o = 2.3 × 10^{-7} mol cm^{-3}) (Huang et al., 2017), υ is the kinetic viscosity of the electrolyte (υ = 8.08 ×10^{-3} cm^2 s^{-1}), k is the electron transfer rate constant, and ω is the rotation speed.

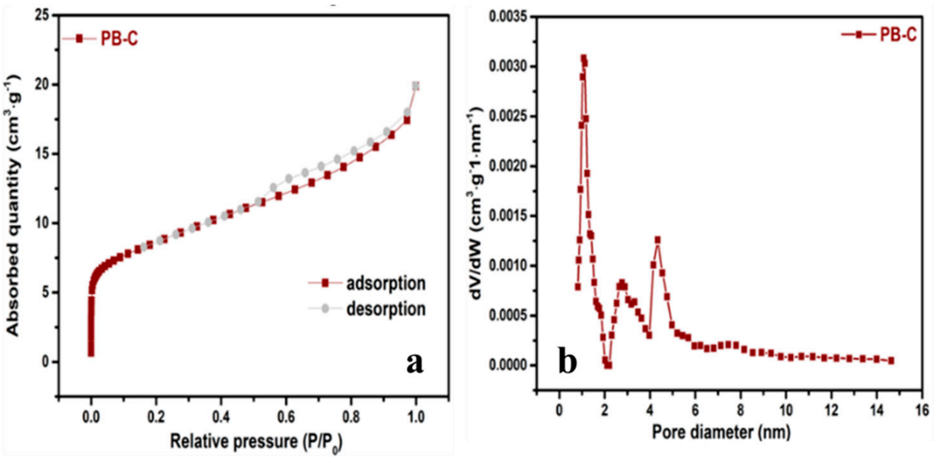

Figure S1 Adsorption and desorption isotherm (a) and (b) pore size distribution of PB-C.

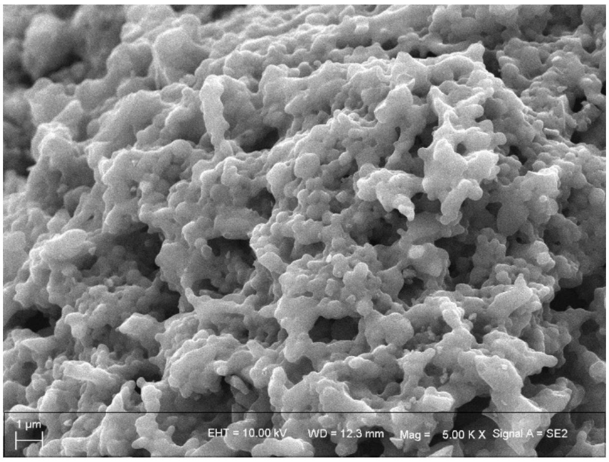

Figure S2 The SEM image of Zn-ZIF-L-derived NC

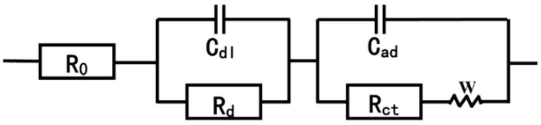

Figure S3. Nernst equation fitting equivalent circuit diagram

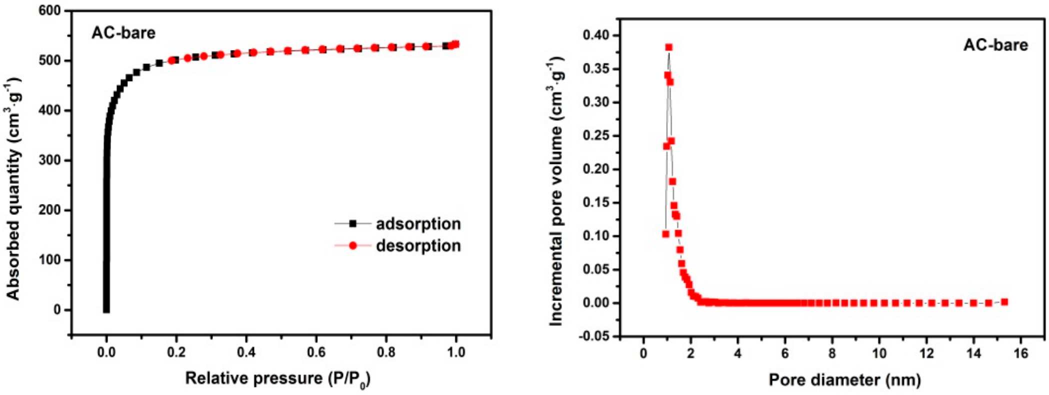

Figure S4. (a,b) Nitrogen adsorption-desorption isotherm and pore size distribution of bared AC.

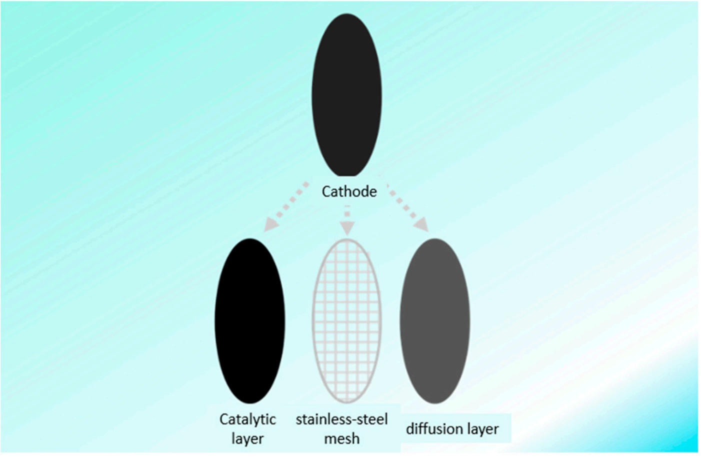

Figure S5 Composition of MFC Cathode

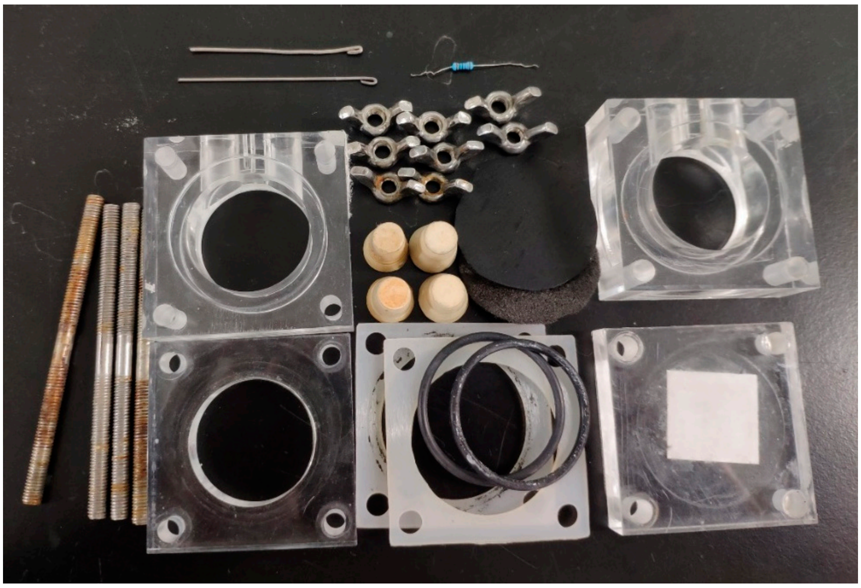

Figure S6 Components of MFC reactor

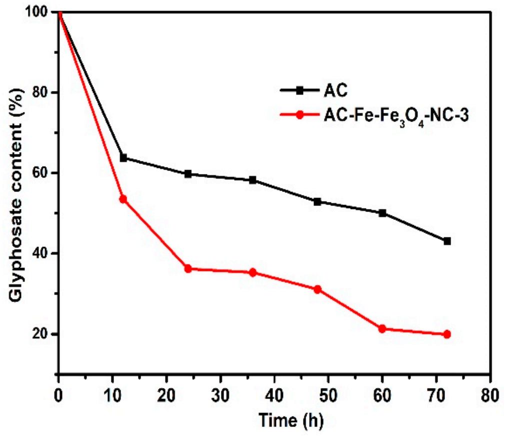

Figure S7 The variation of glyphosate content with degradation time.

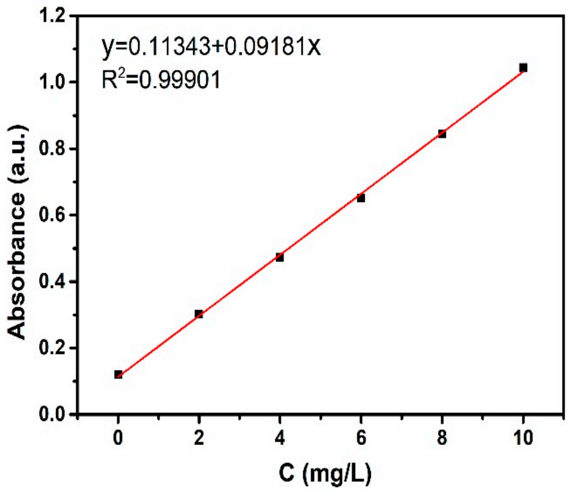

Figure S8 UV-vis calibration curve of the standard glyphosphates solutions.

Table S1 Comparison of surface area and pore information

	**Surface area ($m^2 \cdot g^{-1}$)**	**Total pore volume V ($cm^3 \cdot g^{-1}$)**	**Micropore volume V_{micro} ($cm^3 \cdot g^{-1}$)**	**Mesopore volume V_{meso} ($cm^3 \cdot g^{-1}$)**	**Pore diameter D/nm**
Fe-Fe_3O_4-NC	368.11	0.14	0.0533	0.0823	1.1, 2.3, 4.3
NC	122.90	0.14	0.1282	0.0118	1.5
PB-C	29.97	0.038	0.01343	0.0246	1.1, 2.3, 4.3
